# Gamma Radiation-Induced Effects over an Optical Fiber Laser: Towards New Sensing Applications †

**DOI:** 10.3390/s20113017

**Published:** 2020-05-26

**Authors:** Rosa Ana Perez-Herrera, Andrei Stancalie, Pablo Cabezudo, Dan Sporea, Daniel Neguţ, Manuel Lopez-Amo

**Affiliations:** 1 Department of Electrical Electronic and Communication Engineering, Public University of Navarra, 31006 Pamplona, Spain; pacasava9@gmail.com (P.C.); mla@unavarra.es (M.L.-A.); 2 Institute of Smart Cities (ISC), Public University of Navarra, 31006 Pamplona, Spain; 3 National Institute for Laser Plasma and Radiation Physics, Center for Advanced Laser Technologies, RO-07125 Magurele, Romania; andrei.stancalie@inflpr.ro (A.S.); dan.sporea@inflpr.ro (D.S.); 4 Horia Hulubei, National Institute of Physics and Nuclear Engineering, RO-07125 Magurele, Romania; dnegut@nipne.ro

**Keywords:** erbium-doped fiber amplifiers, optical fiber lasers, laser applications, laser stability, optical fiber sensors, gamma radiation

## Abstract

In the present work, the effect of gamma radiation on the performance of different types of erbium-doped fibers (EDFs) when they are used in a fiber ring cavity (FRC) configuration is studied. Several pieces of commercial EDF are gamma-ray irradiated with different doses to evaluate the output power variations over time. The influence of different doses, from 150 Gy to 1000 Gy, over the output power level measurement and their amplified spontaneous emission (ASE) are experimentally evaluated both in the C and L bands. By using an FRC configuration we can detect the presence of gamma radiation. We can also estimate the irradiation doses applied to EDFs by measuring the slope of the short-term emission power.

## 1. Introduction

Optical fiber systems are deployed everywhere, from our homes to nuclear plants to telecommunication satellites. Because of this, radiation effects on optical fiber devices and associated technologies have been studied extensively in the last decades. Nowadays, it is also possible to include single-mode optical fibers (SMFs) as a sensing solution in large facilities, such as CERN-Large Hadron Collider (LHC) [1], TESLA Test Facility at DESY Hamburg (DESY TTF) accelerator [2], International Thermonuclear Experimental Reactor (ITER) [3,4], Laser Mega Joule (LMJ) [5], for medical radiation dosimetry [6], as well as in modern telecommunication satellites [7].

Optical fiber sensors are now a consolidated technology both in their point or distributed versions. Each approach has its own pros and cons. Both sensing solutions have very interesting characteristics: lightweight, resistance to high temperatures, ability to provide information about temperature or strain over long distances [8], or electromagnetic interference immunity. Thus, research and industry show a higher interest in this technological field. As a result, the optical fiber sensors’ market size is expected to rise to 9.1 Billion USD by 2025, as shown in some reports [9].

When coming to use optical fiber sensors, particularly fiber Bragg gratings (FBGs) in radiation environments, some key aspects must be considered. Due to the continued development of optical fiber manufacturing technologies, radiation hardness becomes a critical parameter in such applications. The most well-known effects are related to radiation-induced attenuation (RIA), due to point defects that create absorption bands [10,11,12,13]. Therefore, radiation-resistant fibers are lately becoming a hot topic in both the academic world and industry [14,15,16,17].

Temperature and strain mapping is a basic feature of optical fiber sensors. This application is especially appealing in radiation environments (e.g., satellite thermal management) [7], structural health monitoring of power grids [18], the nuclear industry (power plants, structural deformations, and vibration detection in reactors) [19], or in the field of medicine (radiation dose monitoring in therapy) [20]. For space applications, doses as high as 300 Gy need about 10 years to be reached. In medical applications, hundreds of Greys are not common for tumor treatment, and the Co-60 is considered the radiation standard for component testing or radiotherapy equipment calibration. This is due to the well know energy levels of the Co-60, to its penetrating power and its homogeneity.

So passive single-mode optical fibers are broadly used as sensing elements. However, for three decades, active optical fibers have been commercially available. One of the major fields of application of active doped optical fibers is their employment inside optical fiber lasers as a gain medium. It is well known that erbium-doped fibers amplifiers (EDFA) are damaged by ionizing radiation. Although their radiation tolerance improvement has been investigated [21,22], they still present a strong degree of degradation, at least in terms of RIA [23,24,25]. Optical amplifiers are being used as well in space-born applications [26], for instance, as a part of transmitters, receivers, satellite telecom, or remote sensing systems. Along with ytterbium-doped fiber amplifiers, EDFs based amplifiers (EDFAs) are optimal amplifiers candidates for those sensing applications.

The radiation-induced impact on EDFAs is strongly dependent on the optical pump wavelength due to the bleaching effects. It was found that the slope efficiency of an optical fiber laser based on an irradiated EDF quickly grows under the action of pumping, owing to the photobleaching of radiation-induced color centers [27], especially at around 980-nm pump wavelength. However, to the best of our knowledge, a direct correlation between accumulated radiation dose, pump power level, optical pump wavelength, and the fiber laser output power level within a defined setup has not yet been reported. Likewise, it could provide important insights into the behavior of such a laser in a radiation environment.

Authors have previously reported some preliminary obtained results from EDF types M12 (980/125) and I25 (980/125) [28,29,30], both designed for small package size C-band amplifiers. The attenuation rate of these irradiated erbium-doped fibers (IEDFs) has also been checked by using an optical backscatter reflectometer (OBR), showing that this characterization agrees well with the measured rise time of the output power level when using them as a gain medium. These OBR measurements revealed increasing attenuation slopes depending on the irradiation doses over the EDFs. According to these results, by using a fiber ring cavity (FRC) configuration, we can detect, in real-time, the presence of radiation and also estimate the irradiation doses applied over the EDFs only by measuring the slope of the short-term emission power. In these previous studies, doses up to 500 Gy were experimentally analyzed.

This paper presents an attempt to improve the experimental analysis of these types of IEDFs, covering two main issues. First, by establishing the gamma radiation influence over a set of EDFs employed as a gain medium in an FRC configuration. Second, by assessing the direct correlation between the gamma radiation doses and fiber lasers performance. For this second issue, a wide range of factors, such as EDFs type and length, pump power level, time of pumping, or even the central emission wavelength or the obtained output power laser stability were taken into consideration. Radiation-induced effects are here evaluated in real-time, by means of an FRC configuration, while lasing through several sets of IEDFs.

## 2. Experimental Setup

This work has been divided into two main stages. In the first one, several sets of EDFs produced by the FiberCore company, (M12 (980/125) and I25 (980/128)), were irradiated with gamma-ray starting from 150 Gy up to 1 kGy and analyzed prior and post irradiation. One reference set of the same fibers was kept for comparison.

The selected type of these EDFs, which offer a wide range of absorption rates, cladding diameters, and pump wavelengths, allowed attaining the lasing in the C-band and the L-band. In the specific case of fibers with a high erbium-doped concentration, such as the M12 and I25 type, they were generally used for the L-Band, although the length of the fiber may also vary the peak wavelength to a certain extent [31]. Model M12, with an absorption loss of about 12 dB/m at 980 nm, has an estimated dopant concentration of $3.535\cdot 10^{25}$ ions/m^3^ [31]. On the other hand, model I25 presents an absorption loss of 24 dB/m at 980 nm, which can be translated to an estimated erbium ion concentration doping of 2000 ppm [32,33]. Table 1 resumes some of the main properties of these two samples.

As it has previously pointed out in [34], EDF length can be optimized to minimize gain fluctuation, that is, to provide a wider flat gain spectrum. Therefore, considering a number of factors, such as erbium ion density, numerical aperture, or manufacturing losses, the optimized length for this purpose, were calculated to be around 5 m length [34].

The irradiator was a GC-5000 Co-60 gamma chamber (B.R.I.T., Mumbai, India) and the dose rate was 5.7 kGy/h (+/− 1.8%). The fibers were placed centrally in the irradiation chamber, symmetrically with respect to the cylindrical gamma source, so that the irradiation was as homogenous as possible. Alanine dosimeters were utilized to check the homogeneity of the radiation field and the final accumulated dose. The dosimetry system is traceable to National Metrology Institute-NPL (Teddington, UK) and Risø High Dose Reference Laboratory-H.D.R.L. (Denmark). The marked position was kept as a reference for each fiber that was irradiated so that all pieces were placed in the same spot inside the chamber. After the irradiation, all fibers were checked via an optical frequency reflectometer (OBR 4600 from LUNA Inc., Roanoke, VA 24011, USA) to clearly highlight RIA as a factor of the total accumulated dose for each IEDF type.

**Figure 1:**
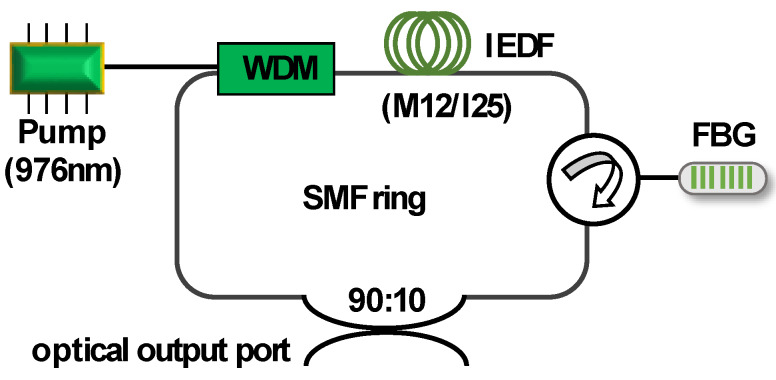
Schematic of the fiber ring cavity configuration, where several irradiated erbium-doped fibers (IEDFs) were used as an active medium.

In the second stage, the FRC configuration was experimentally implemented. Figure 1 shows a schematic diagram of the experimental setup used to evaluate the laser generation properties when using these types of IEDFs as an active medium within the cavity. A 980/1550 nm wavelength division multiplexer (WDM) injected the pump laser centered at 976 nm into the FRC configuration, and the studied IEDF samples were located at the common port of this multiplexer. To insert the intended wavelengths into the FRC configuration, a uniform FBG centered on 1550.7 nm with an optical circulator was used as a feedback element. The use of an optical circulator also minimized the undesired hole burning effect. In addition to this, a 90% optical coupler was used to extract about 10% of the laser output power from the ring to be connected to an optical spectrum analyzer (OSA) with an optical resolution of around 0.03 nm and controlled via MATLAB software, so that all the data were recorded with a given acquisition rate. The experimental studies were carried at constant room temperature.

The IEDFs utilized as an active medium in the setup were investigated independently, for each gamma radiation dose. For every experimental test carried out, pump power was increased gradually from 100 mW to 250 mW. Before evaluating the output power level obtained by using these IEDFs, a reference sample of non-irradiated EDF of both types was employed, for a comparative reason. 

The FRL configuration, including the IEDFs, was pumped from 2 h up to 135 h, depending on the gamma radiation dose, to understand better the irradiation effects as a factor of photobleaching on these highly doped fibers.

Our main goal was to provide a better insight into the dependence between RIA effects on the active medium and the measured output power levels for possible sensing applications in extreme environments. This experimental study opens the door to having a simple and effective system that provides data about the irradiation level, by correlating irradiation effects with the obtained values (in terms of output power rising time, pump power levels, or pumping time.) Thus, the experimental setup was designed to be flexible enough to assess both permanent and temporary radiation-induced effects.

## 3. Results

**Figure 2:**
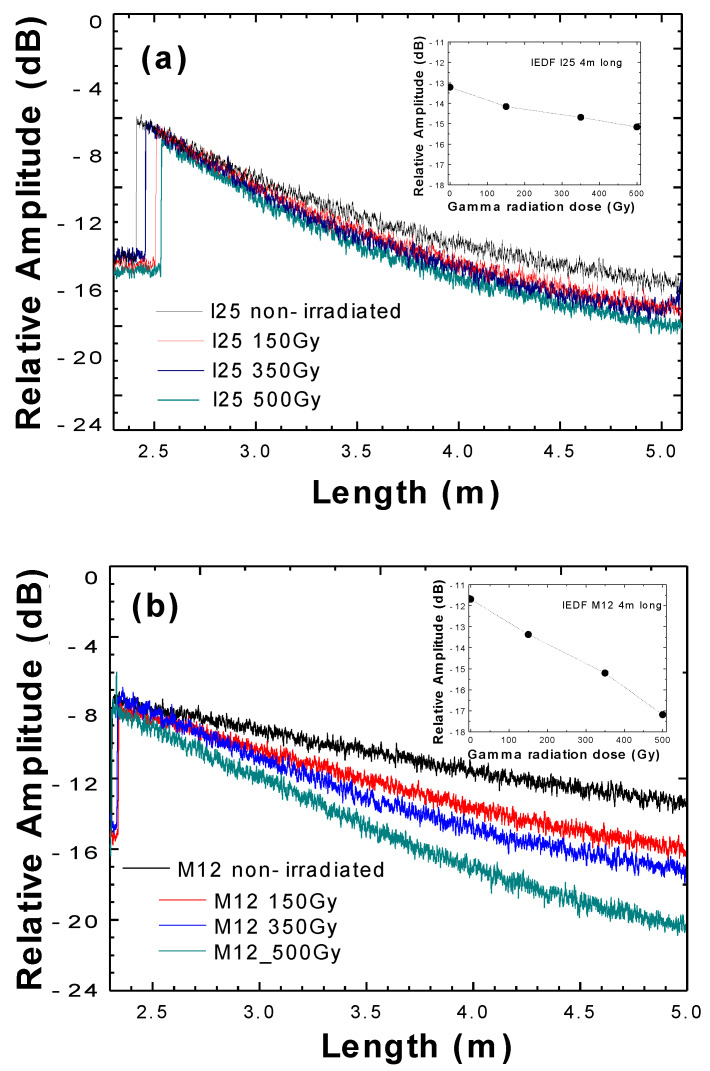
Backscattered optical power of the IEDF type (**a**) I25 and (**b**) M12 as a function of their length for gamma radiation doses of non-irradiated (black), 150 Gy (red), 350 Gy (blue), and 500 Gy (green). Insets: backscattered optical power as a function of gamma radiation doses for a representative length of 4 m long of I25 (inset Figure 2a) and M12 (inset Figure 2b).

To investigate radiation-induced effects independently, before splicing the irradiated EDFs into the fiber ring configuration, their optical transmission was measured with an optical frequency domain reflectometer (OFDR) and compared with reference (non-irradiated) fibers. Figure 2 presents the backscattered optical light dependence to accumulated gamma radiation dose as a function of their length, showing a good correlation between both parameters. These experimental results were obtained six months after gamma irradiation. During this time, the samples were kept at constant room temperature to anneal the unstable radiation defects. Therefore, the following data we obtained focuses on photo-annealing only, based on the recovery of other temporary radiation-induced effects. In this manner, the proposed solution proves to be viable a long time after the irradiation. 

The variation of the backscatter power (measured in dB) is not linearly dependent on the applied dose, as Figure 2 also represents. However, here it is experimentally demonstrated that a relation exists since optical transmission decreases with radiation dose and gamma radiated fiber length. The higher the applied gamma dose, the higher the radiation-induced attenuation [10]. The inset of these figures also shows the backscattered optical power as a function of gamma radiation doses for a representative length of 4 m long of each IDEF type I25 and M12 in that order.

**Figure 3:**
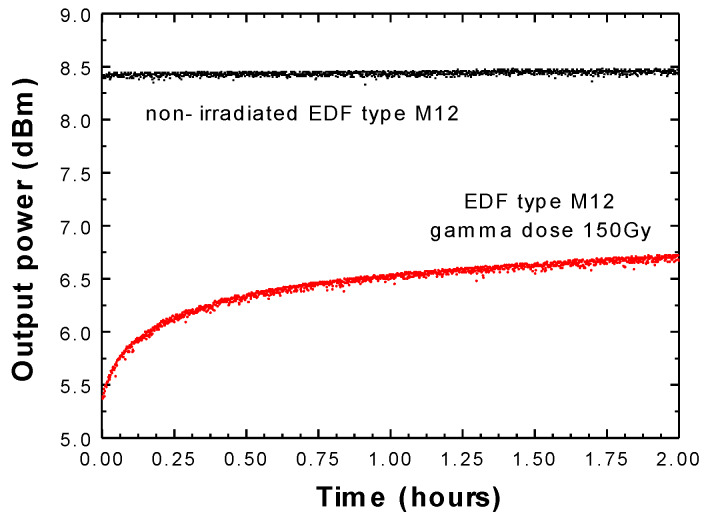
Output power variation over time when using 5 m of a non-irradiated EDF M12 (black) or an IEDF M12 with 150 Gy (red).

**Figure 4:**
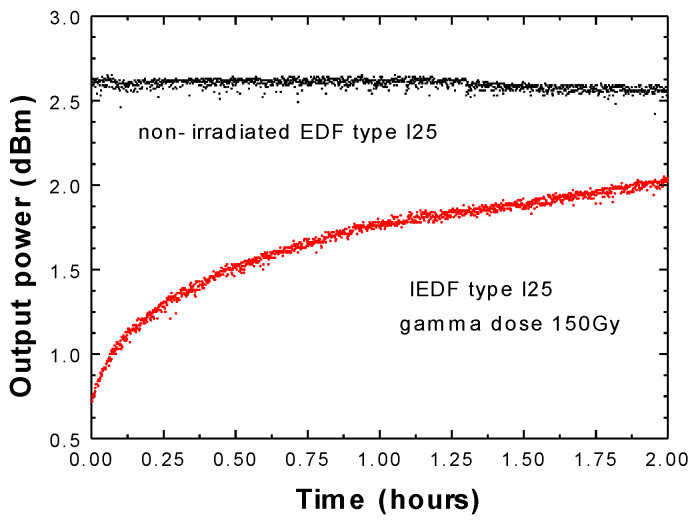
Output power variation over time when using 5 m of a non-irradiated EDF I25 (black) or an IEDF I25 with 150 Gy (red).

In the subsequent step, a non-irradiated EDF sample of each type was used as an active medium into the FRC to estimate the output power level stability experimentally. On the other hand, when IEDFs were evaluated, it was found that the output power level did not remain as stable. To better exemplify, Figure 3 and Figure 4, present the output power variation as a function of time in the case of IEDF type M12 (Figure 3) or IEDF type I25 (Figure 4), both irradiated with 150 Gy (red), compared with the flat response over time obtained by using a non-irradiated EDF sample (black). These measured output power levels, when pumped by a 976 nm laser at 100 mW and using 5 m long non-irradiated EDF (black) or IEDFs (red) as the active medium, were evaluated with an acquisition rate of 5 s for 2 h total.

The rise in the output power level was demonstrated to be predominant in the first hour of pumping, as Figure 3 and Figure 4 illustrate. Consequently, the time required for the output power level to increase from 10% to 90% of its final value was valued for this type of IEDF. As a result, the attained rise time for the IEDF M12 and I25, both for gamma radiation dose of 150 Gy was 36.41 min (see Figure 3), and 42 min (see Figure 4) in that order.

As it is presented in this work, the higher the total accumulated dose, the longer the rise time. Therefore, those values can be used not only to discriminate the presence of gamma radiation but also to estimate the applied dose. 

To better explain the loss and recovery mechanism for the EDFs, we need to understand the changes in the macroscopic properties of the silica fibers that are induced by gamma radiation. The most important mechanism to consider, along with refractive index change, is the radiation-induced absorption. This occurs mostly by the generation of point defects in the fiber’s core or cladding due to the ionization of displacement damages manifesting in a decrease in in-fiber transmission. Ionizing radiation leads to structural alteration in the pure or doped amorphous silica matrix. The resulting optical absorption bands further lead to the observed optical power loss of around 3 dBm, respectively, 2 dBm for the two types of fibers under test (M12 or I25, respectively), when subjected to a dose of 150 Gy. The difference between the effects of the two fibers is explained by the host glass matrix. However, because of proprietary design information, we are not allowed to present here some of the fiber’s chemical composition.

On the other hand, although the photo annealing effect of a 976 nm source over EDF is considered somehow complicated [27] due to (a) Er3+ absorption of 976 nm light and temperature rise in the fiber’s core; and (b) possible excitation of 532 nm due to up-conversion of the Er3+, our experimental results show good agreement between 976 nm photobleaching time and the recovery of gamma radiation induced absorption into the two EDFs tested. Due to the period between the irradiation of the fibers and the reported results, we conclude the photobleaching process enabled the partial deexcitation of the high energy levels of the electrons absorbed by the outer orbital electrons of the Er3+, to the ground state or to an intermediate level. Second, the deexcitation may be a result of the photons provided by the bandgap between intermediate to ground state, leading to an amplification of the optical signal.

However, the limitations of doses higher than 750 Gy are strongly noticed, making the configuration convenient for sensing in low dose applications. Moreover, as the subject of exploring EDF under irradiation is intensively reported in the literature [35,36,37,38], we would like to focus as a novelty on the performance of a fiber laser having irradiated components as active elements as well to open the possibility to address a new radiation sensing configuration.

Once this influence of the gamma IEDF over the output power level was experimentally demonstrated, a more detailed study both in terms of amplified spontaneous emission (ASE) and a deeper output power level analysis over time, was carried out.

### 3.1. Amplified Spontaneous Emission (ASE)

As is well known, the spectral shape of the amplified spontaneous emission (ASE) is strongly dependent on several parameters, such as fiber length, doping dose, or the pump power level, among others. For that reason, each radiation dose and each type of EDFs provide a different spectral figure of their ASEs. Consequently, different wavelength emission lines could be attained when inserting these irradiated gain media into a cavity ring laser.

**Figure 5:**
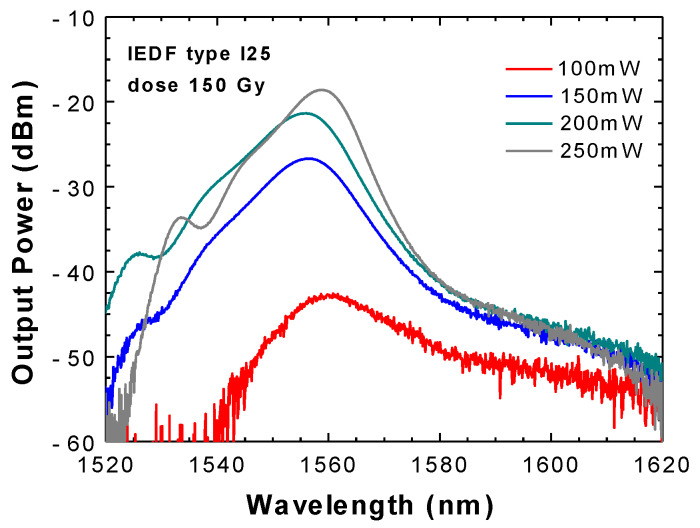
Amplified spontaneous emission (ASE) spectra obtained from a 5 m length of EDF I25 irradiated with 150 Gy when pumped by a 976 nm light source from 100 mW to 250 mW.

Figure 5 shows the exit ASE spectra measured in the amplifier configuration, that is when the free end of the IEDF was connected directly to the OSA. In this case, 5 m of IEDF I25 irradiated with a gamma dose of 150 Gy were pumped from 100 mW to 250 mW.

**Figure 6:**
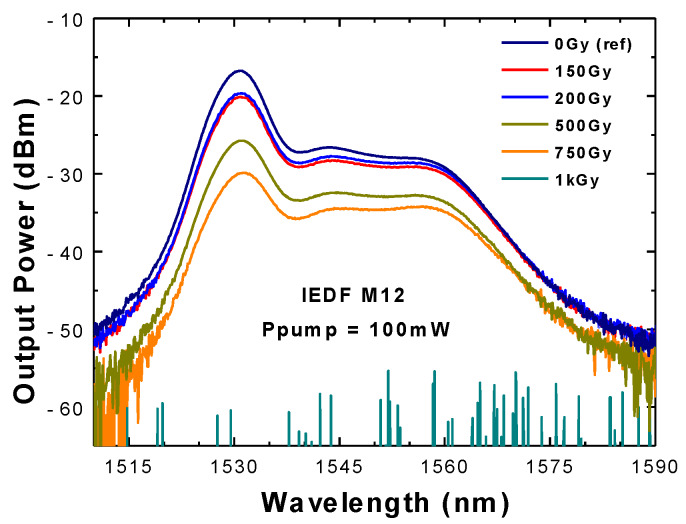
ASE spectra obtained from a 5 m length of EDF M12 irradiated with different doses from 0 Gy (reference) up to 1 kGy when pumped by a 976 nm light source with 100 mW.

The ASE spectra were also evaluated for 5 m length of IEDF type M12, irradiated with doses up to 1 kGy of gamma-rays, when pumped by a 976 nm light source with 100 mW power. Very different shapes of the exit ASE spectra were measured, as can be seen in Figure 6.

More than 13 dB optical loss was measured when comparing the output power level around 1530 nm for the non-irradiated EDF, used as a reference, to the one irradiated with 750 Gy. Furthermore, there was no visible ASE spectrum when a 1 kGy gamma IEDF was connected to the OSA due to its high level of RIA (black line shown at the bottom of Figure 6).

This work focused on analyzing the ionizing radiation effect (in current case gamma-ray) on different types of EDFs when they were employed into an FRC configuration. A flat region of the erbium gain profile was selected for the FBG´s central wavelength emission (1550.7 nm), to minimize undesired output power level fluctuations [29,30]. Hence, a thorough study was carried out by using the IEDF type M12 and not the I25. That decision was based on several factors: first, I25 ASE spectra lacked a flat region, and also, it has been recently demonstrated that I25 presents high gain spectrum fluctuations as a function of temperature variations [34]. Finally, M12 was chosen in this deeper analysis because its gain fluctuations were in the average value for all types of EDFs, as is presented in [34].

### 3.2. IEDF M12 over Time

The next step consisted in the evaluation of each gamma IEDF type M12, as dependent on accumulated doses, using the implemented setup presented in Figure 1. The acquisition rate was of 5 s until the complete stabilization of the output power level. For this to be accomplished, a part of the samples needed at least 24 h for pegging this value.

The absolute output power level can be vaguely altered by the quality of the splices carried out to insert the IEDFs into the cavity of the ring laser. For that reason, special attention was directed to the slightest variations over the peak power levels for each sample subject of study.

**Figure 7:**
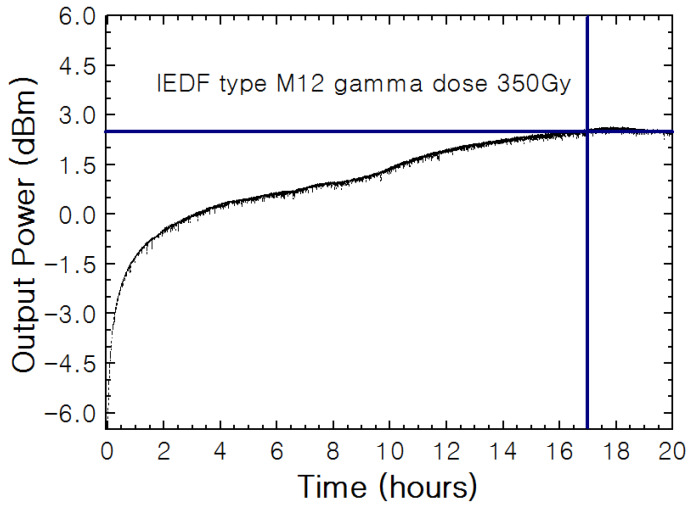
Fiber laser output power variation over 20 h when using 5 m of EDF M12 irradiated with 350 Gy when pumped by a 976 nm laser at 100 mW.

**Figure 8:**
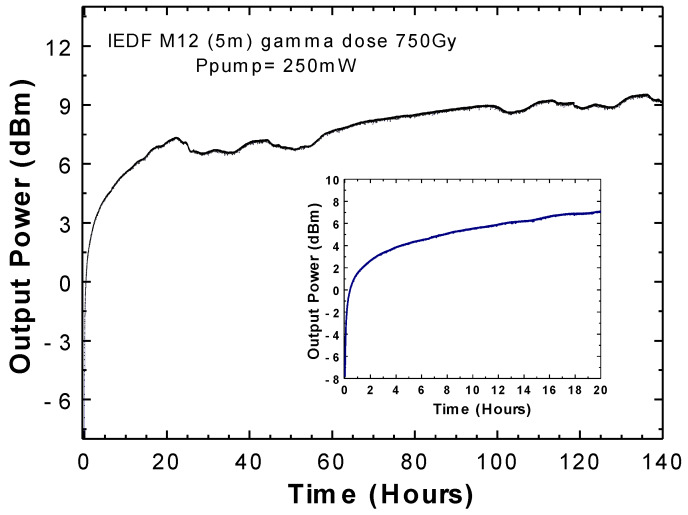
Output power variation over 140 h when using 5 m of an IEDF M12 with 750 Gy when pumped with 250 mW. Inset: detail of the first 20 h of pumping.

Figure 3 illustrates the output power variation over time when using 5 m of IEDF M12 irradiated with 150 Gy. In that case, 2 h were required to reach the stabilized output power level. The period of time to stabilize this output power level was expected to be longer when higher doses of radiation were applied to the EDFs samples. That was experimentally demonstrated when two samples of IEDF type M12 irradiated with higher gamma radiation dose were used as gain media. Figure 7 and Figure 8 show the output power variation over time when using IEDF type M12 irradiated with 350 Gy and 750 Gy, respectively, where around 17 and about 100 h in that order were needed to reach a stable output power value. These experimental results demonstrate that the duration for stabilizing the output power level is directly dependent on radiation dose.

As mentioned above, Figure 7 illustrates the output power variation over 20 h when using 5 m of an IEDF type M12 irradiated with 350 Gy. As shown, about 17 h were necessary for this value to remain constant. As in previous tests, the more noteworthy rise of the output power level emerged during the first hours of pumping, as is shown in this figure. In this case, the rising time for a gamma radiation dose of 350 Gy was 43 min, which means an increase in the rise time of about 18% when compared with a dose of 150 Gy.

We would like to mention that the slight variation shown in the last two hours of the experimented was correlated with a decrease in room temperature during the night. However, for accuracy sake, we decide to report all the recorded experimental data.

Finally, a higher dose of gamma-radiation was applied to a new fiber sample with 5 m length of EDF M12. As previously demonstrated, a reduction of about 13 dB of gain at around 1530 nm was measured when compared to the ASE spectrum for the non-irradiated fiber with the IEDF with the 750 Gy. That means that the pump power needed for attaining a laser wavelength emission should be higher than in our previous experiments. In this configuration, 48 h of pumping with 250 mW, instead of 100 mW, was necessary for the laser emission line to emerge. After that moment, the output power laser behavior over time, was the one presented in Figure 8, showing that around 100 h of pumping was required to reach a stable output power value of 9 dBm. It is worth noticing that, in this longer experimental study, room temperature fluctuations induced minor output power variations.

In the inset of Figure 8, the first 20 h of pumping are also illustrated to best compare with data shown in Figure 7. Here, we report experimentally a rising time of about 230 min, in correlation to a gamma radiation dose of 750 Gy, corresponding to an increment of more than 430% when compared to an EDF irradiated with 350 Gy.

The experimental results confirmed the initial hypothesis that the higher the gamma doses, the higher the rising time. Analysis of the reported values can be used to discriminate the presence of gamma radiation and, also to estimate the applied doses over the EDF.

To sum up, in this experimental study, the gamma radiation effects were investigated in terms of (a) RIA effects (by using an OBR); (b) evaluating the output power level variation over time; and (c) considering the different ASE spectra measured when using IEDFs as a gain media irradiated with gamma doses from 150 Gy up to 1 kGy. It has also been experimentally demonstrated that employing EDFs irradiated with more than 500 Gy as an active medium into the cavity ring laser, the corresponding emission wavelength emerged only after being pumped for several days. Therefore, we find it appealing to further analyze the pump time/power dependence of such an active medium. 

The advantages of such an approach are as follows:

1. The measurements can be performed a long time after the irradiation has taken place, as no photo-annealing was noticed after a few months;
2. Due to the fact that the RIA level decreases with pump time, most of the IEDF may be re-utilized, making the system even more cost-effective;
3. The device has a low price and can be integrated into real-time monitoring systems, using not only optical fiber sensors but also EDFs as sensing elements.

## 4. Conclusions

In the current work we presented, for the first time to the best of our knowledge, the gamma radiation effects on the output power level of a fiber ring cavity configuration, when utilizing IEDFs as an active medium. Besides analyzing the behavior of such laser parameters, as a factor of total accumulated dose, we reported on the proof-of-concept experiment dedicated to the investigation of a new optical fiber radiation sensor. In this case study, only the EDF samples were irradiated. We report on two types of commercial EDFs irradiated with different gamma radiation doses to study the output power variations over time. Total doses from 150 Gy up to 1 kGy were applied to EDFs. The impact of gamma radiation over the attained output power level measurement and the ASE was experimentally evaluated, after analyzing RIA by means of an optical backscatter reflectometer. We found experimentally that, by using a fiber ring laser cavity configuration, it is possible to detect the presence of radiation. Driven by a lower cost solution, possible targeted applications include radiation monitoring for access points of personnel in nuclear facilities, space applications, or medical radiation therapy. In addition, considering the obtained results, by measuring the optical emission rising time, that is, the slope of the short-term emission power evolution, an estimation of the irradiation doses applied to the samples is possible. The functioning regime of such a laser was analyzed comparatively, with two types of non-irradiated erbium-doped fibers, giving insights gradually on the radiation-induced effects.

## Figures and Tables

**Table 1:** Main characteristics of tested highly doped erbium fibers [34].

EDF	Absorption at 980 nm (dB/m)	Absorption at 1531 nm (dB/m)	Numerical Aperture	Ion Density (ppm)	Core Concentricity (µm)	Coating Diameter (µm)	Cladding Diameter (µm)	Cut-Off Wavelength (nm)
M-12	11–13	16–20	0.21–0.24	900	≤0.3	245 ± 15	125 ± 1	900–970
I-25	24	35–45	0.23–0.26	2200	≤0.5	245 ± 7	125 ± 1	900–970
